# Association of sarcopenia and physical activity on the severity of metabolic dysfunction-associated steatotic liver disease among United States adults: NHANES 2017 - 2018

**DOI:** 10.3389/fragi.2025.1573170

**Published:** 2025-05-13

**Authors:** Xiaodie Wei, Xiaohui Liu, Jinhan Zhao, Yang Zhang, Lixia Qiu, Jing Zhang

**Affiliations:** ^1^ The Third Unit, The Department of Hepatology, Beijing Youan Hospital, Capital Medical University, Beijing, China; ^2^ Beijing Institute of Hepatology, Beijing Youan Hospital, Capital Medical University, Beijing, China

**Keywords:** metabolic dysfunction-associated steatotic liver disease, physical activity, sarcopenia, NHANES, liver fibrosis

## Abstract

**Background:**

Sarcopenia, physical activity (PA), and sedentary behavior are associated with metabolic dysfunction-associated steatotic liver disease (MASLD). The study aimed to evaluate the effects of sarcopenia and PA on the presence and severity of MASLD.

**Methods:**

This cross-sectional study analyzed data from the 2017-2018 National Health and Nutrition Examination Survey (NHANES). Hepatic steatosis and liver fibrosis were assessed by vibration-controlled transient elastography (VCTE). Sarcopenia was defined based on the Foundation for the National Institutes of Health criteria. PA and sedentary behavior were evaluated using the Global Physical Activity Questionnaire (GPAQ).

**Results:**

Among 1,831 participants, 664 were diagnosed with MASLD, including 482 with severe steatosis and 89 with significant fibrosis. The prevalence of sarcopenia in the MASLD and non-MASLD populations was 11.7% and 3.8%, respectively. Multivariable-adjusted models demonstrated that sarcopenia significantly increased the risk of MASLD (OR 2.45; 95% CI: 1.33–4.52), severe steatosis (OR 2.56; 95% CI: 1.40–4.66), and significant fibrosis (OR 6.10; 95% CI: 2.08–17.84). Additionally, individuals with sarcopenia and low PA had a 7.91-fold increased risk of developing significant fibrosis (OR, 7.91; 95% CI: 1.42–44.16, P = 0.022). Sarcopenia and prolonged sedentary behavior further increased the risk of MASLD (OR 3.75; 95% CI: 1.60–8.76), severe steatosis (OR 17.58; 95% CI: 1.93–159.79), and significant fibrosis (OR 4.32; 95% CI: 1.31–14.31).

**Conclusion:**

Patients with sarcopenia should increase physical activity and reduce sedentary time to decrease the risk and progression of MASLD. Increasing muscle mass and strength through resistance exercise to reduce the risk of significant fibrosis in sarcopenia patients.

## 1 Introduction

Metabolic dysfunction-associated steatotic liver disease (MASLD) has emerged as the most rapidly increasing chronic liver disease worldwide, affecting more than 30% of the global population (Miao et al., 2024; Younossi et al., 2024). MASLD encompasses liver conditions ranging from metabolic dysfunction-associated steatotic liver (MASL), metabolic dysfunction-associated steatohepatitis (MASH), liver fibrosis, and cirrhosis (Targher et al., 2024). The presence of MASLD is closely linked to cardiometabolic risk factors like obesity, type 2 diabetes, and metabolic syndrome (Targher et al., 2021). Sarcopenia, as defined by the Global Leadership Initiative on Sarcopenia (GLIS), is a progressive and systemic skeletal muscle disease characterized by a reduction in both muscle mass and strength (Kirk et al., 2024). Notably, sarcopenia and MASLD may exacerbate each other’s progression through shared mechanisms, including insulin resistance, chronic inflammation, and hormonal imbalance (Merz and Thurmond, 2020; Altajar and Baffy, 2020). The process of muscle loss results in changes to muscle-related factors, such as elevated myostatin and reduced irisin levels, which exacerbate insulin resistance and systemic inflammation via the endocrine effects, influencing the progression of metabolic diseases (Polyzos et al., 2023).

Physical activity (PA) also plays a critical role in preventing and managing sarcopenia and MASLD, enhancing metabolic health, muscle mass, and function (Piercy et al., 2018; Jamali et al., 2022). PA has been proven effective in preventing MASLD and delaying its progression. Notably, engaging in moderate to vigorous PA was associated with a reduced risk of mortality in patients with MASLD (Golbidi et al., 2012; Kim et al., 2021). Furthermore, prolonged sedentary behavior significantly increased the risk of developing diabetes and cardiovascular diseases (Grøntved and Hu, 2011). PA guidelines emphasized that reducing sedentary behavior can benefit nearly everyone (Piercy et al., 2018). It has been proven that sarcopenia, physical inactivity, and prolonged sedentary behavior were risk factors for MASLD (Kim et al., 2022; Chun et al., 2023; Kim et al., 2020; Harring et al., 2023). Importantly, individuals with sarcopenia often have less PA, potentially further exacerbating their risk for liver steatosis and fibrosis. Despite increasing evidence for the independent effects of sarcopenia and physical activity on MASLD, the combined influence of these factors on disease severity remains poorly understood (Kim et al., 2020; Harring et al., 2023; Franco et al., 2024; Golabi et al., 2020). It is essential for developing integrated strategies to mitigate the burden of MASLD in individuals with sarcopenia.

Therefore, this study aimed to explore the association between sarcopenia, physical activity, and MASLD severity using data from the NHANES (National Health and Nutrition Examination Survey). We hypothesized that physical inactivity and prolonged sedentary behavior increase the risk of MASLD and liver fibrosis in individuals with sarcopenia.

## 2 Methods

### 2.1 Subjects and study design

This retrospective study analyzed the data from NHANES 2017-2018, which employed a national, multistage, and stratified sampling design to obtain a representative sample of the U.S. population. NHANES 2017-2018 was the first to use Fibroscan measurements of CAP and VCTE to assess liver steatosis and fibrosis. This approach was adopted due to the extensive evaluation of elastography for its accuracy in assessing hepatic steatosis and fibrosis. MASLD was diagnosed based on the EASL-EASD-EASO Clinical Practice Guidelines, which require the presence of hepatic steatosis through imaging techniques (vibration-controlled transient elastography), combined with at least one of the five defined cardiometabolic risk factors (Rinella et al., 2023). Hepatic steatosis was quantified using the controlled attenuation parameter (CAP), with steatosis defined as a median CAP value of ≥248 dB/m. Moderate and severe steatosis were further classified with CAP values of ≥268 dB/m and ≥280 dB/m, respectively (Tracker, 2024). Significant liver fibrosis was defined as a median liver stiffness measurement (LSM) of ≥8 kPa. The NHANES study was ethically approved by the Ethics Review Board of the National Center for Health Statistics, and all participants provided written informed consent.

### 2.2 Clinical and laboratory assessments

Variables were categorized using previously established methods. Race was divided into five groups: non-Hispanic white, non-Hispanic black, Hispanic, non-Hispanic Asian, and others. Educational status was classified as college or non-college graduate, while marital status was categorized as married or other. Smoking status was defined as never smoked, former smoker, or current smoker.

Body Mass Index (BMI) was calculated by dividing weight (kg) by height squared (m^2^). Obesity was defined as a BMI >25 kg/m^2^ for Asians and >30 kg/m^2^ for other races (WHO Expert Consultation, 2004). Hypertension was defined as a systolic blood pressure of ≥140 mm Hg, a diastolic blood pressure of ≥90 mm Hg, and current treatment with antihypertensive medications (Whelton et al., 2018). Diabetes mellitus was defined as a fasting blood glucose level of ≥126 mg/dL, glycated hemoglobin (HbA1c) of ≥6.5%, or the use of hypoglycemic drugs or insulin (American Diabetes Association, 2018).

### 2.3 Sarcopenia definition

Sarcopenia was defined using appendicular lean mass (ALM) measured by dual-energy X-ray absorptiometry (DXA), which quantifies lean mass in all four limbs to determine total ALM. The diagnostic criteria for sarcopenia followed the guidelines of the Foundation for the National Institutes of Health (FNIH), defined as an ALM/BMI <0.789 for men and <0.512 for women (Studenski et al., 2014).

### 2.4 Physical activity

Detailed physical activity (PA) data were collected using the global physical activity questionnaire including leisure-time physical activity (LTPA), work-related physical activity (WPA), transportation-related physical activity (TPA), and sedentary behavior (sitting time) (Armstrong and Bull, 2006). Each type of PA was assessed for weekly frequency (days per week), duration (minutes), and intensity (vigorous vs. moderate). Total PA time was calculated as the sum of vigorous activity time and 2 times spent in moderate activity. The total PA was defined as the total of LTPA, WPA, and TPA. According to the 2018 guidelines, PA was categorized into 0-149 min/week and ≥150 min/week (Piercy et al., 2018). To investigate sedentary behavior, we assessed total sitting time by the reported hours per day in a typical week. Sedentary behavior was defined as total sitting time≥ 420 min/d (Kim et al., 2022).

### 2.5 Statistical analysis

All analyses accounted for the weights of each variable provided in the NHANES database, given the complex survey design of NHANES. Continuous variables were described as weighted means with standard error (SE), and categorical variables were presented as weighted frequencies with weighted percentages. Multivariate logistic regression models were used after adjusting for potential confounding factors, including age, sex, race, education, marital status, smoking status, T2DM, hypertension, hyperlipidemia, ALT, AST, triglycerides, and total cholesterol. Missing data in key variables were handled using multiple imputation by chained equations (MICE), a method commonly used for handling missing data in complex survey designs. The extent of missing data in key variables was minimal, with no variable exhibiting more than 6% missing data. All statistical analyses were conducted using R software (version 4.3.3; R Foundation for Statistical Computing, Vienna, Austria), with a two-sided p-value of <0.05 considered statistically significant.

## 3 Results

### 3.1 Baseline characteristics of the study population

**FIGURE 1:**
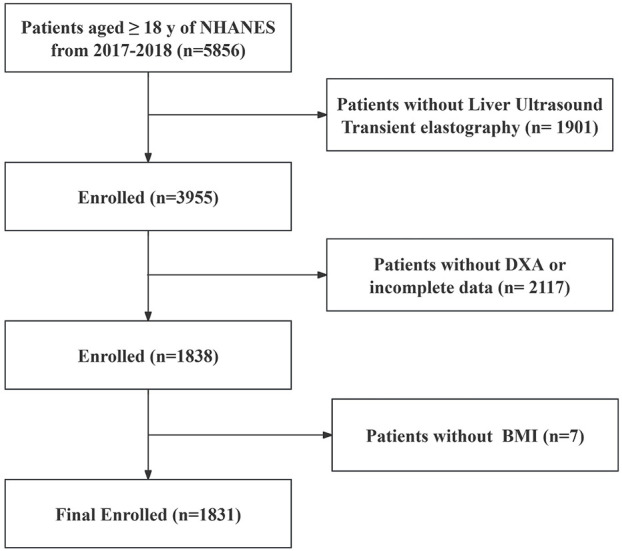
Flowchart of the study population. According to the exclusion criteria of the study, 4,025 adults were excluded from the 5,856 adults, and a total of 1831 patients were finally analyzed. NHANES, National Health and Nutrition Examination Survey; MASLD, Metabolic dysfunction-associated steatotic liver disease; BMI, Body mass index; DXA, Dual-energy X-ray absorptiometry.

After excluding individuals who did not meet the inclusion criteria, 1,831 participants were included in the study (Figure 1). The baseline characteristics were detailed in Table 1. The mean age of the participants was 37 years, and males accounted for 49.18%. The mean BMI was 27.61 kg/m^2^. The majority of the participants were non-Hispanic whites (58.02%) and non-Hispanic blacks (11.67%).

**TABLE 1:** Baseline characteristics of the study population.

Variables	Total (n = 1831)	Non-MASLD (n = 1,167)	MASLD (n = 664)	P Value
Age, y	37.42 (0.59)	34.79 (0.68)	42.80 (0.69)	<0.0001
18-29	33.82 (0.03)	41.65 (2.58)	17.80 (1.76)	
30-44	33.46 (0.02)	34.67 (1.88)	30.99 (2.23)	
45-60	32.72 (0.03)	23.68 (2.21)	51.21 (2.98)	
Male, %	49.18 (0.03)	44.62 (2.28)	58.51 (2.17)	0.002
Race, %				<0.001
Mexican American	9.38 (0.02)	7.60 (1.63)	13.03 (2.99)	
Asian	8.13 (0.01)	6.67 (1.23)	11.12 (2.33)	
Non-Hispanic Black	11.67 (0.01)	12.58 (1.76)	9.80 (1.50)	
Non-Hispanic White	58.02 (0.06)	60.33 (3.20)	53.28 (3.98)	
Other Hispanic	8.43 (0.01)	8.67 (1.19)	7.93 (1.23)	
Other Race	4.38 (0.01)	4.15 (0.66)	4.84 (1.57)	
College education, %	61.83 (0.04)	61.20 (2.84)	63.12 (3.33)	0.59
Married, %	46.22 (0.04)	37.62 (2.41)	63.81 (2.87)	<0.0001
Smoke, %				0.06
Former	18.62 (0.02)	16.37 (2.42)	23.23 (2.93)	
Never	65.78 (0.04)	65.49 (3.25)	66.36 (3.77)	
Now	15.60 (0.02)	18.14 (1.95)	10.40 (1.91)	
Body mass index, kg/m^2^	27.61 (0.33)	25.24 (0.33)	32.46 (0.46)	<0.0001
Waist circumference, cm	93.82 (0.92)	87.25 (0.90)	107.25 (1.18)	<0.0001
Liver stiffness measurement, kPa	5.15 (0.10)	4.69 (0.09)	6.10 (0.23)	<0.0001
Alanine aminotransferase, IU/L	22.33 (0.76)	20.05 (0.90)	26.94 (0.98)	<0.0001
Aspartate aminotransferase, IU/L	21.65 (0.52)	21.44 (0.76)	22.09 (0.58)	0.54
Triglyceride, mmol/L	1.48 (0.05)	1.17 (0.04)	2.11 (0.11)	<0.0001
Total cholesterol, mmol/L	4.78 (0.04)	4.69 (0.05)	4.97 (0.06)	0.001
Fasting glucose, mg/dL	94.70 (1.02)	89.07 (0.40)	106.08 (2.96)	<0.0001
Hemoglobin A1c, %	5.47 (0.03)	5.28 (0.01)	5.86 (0.08)	<0.0001
Sarcopenia, %	6.35 (0.01)	3.77 (0.75)	11.65 (1.82)	<0.0001
Hypertension, %	22.37 (0.02)	14.05 (2.07)	39.41 (2.85)	<0.0001
T2DM, %	7.20 (0.01)	1.63 (0.25)	18.60 (2.60)	<0.0001
Hyperlipidemia, %	52.21 (0.03)	41.44 (2.24)	74.24 (2.47)	<0.0001
Obesity, %	33.38 (0.03)	16.95 (2.44)	67.03 (4.10)	<0.0001
Total physical activity, min/wk	1583.58 (96.52)	1690.85 (105.33)	1339.52 (113.66)	0.002
Work-related physical activity, min/wk	942.79 (85.88)	1027.98 (90.51)	768.44 (106.36)	0.01
Transportation-related physical activity, min/wk	77.42 (6.65)	84.14 (8.39)	63.65 (10.21)	0.13
Leisure-time physical activity, min/wk	308.72 (14.55)	355.09 (17.94)	213.81 (19.70)	<0.0001
Sedentary activity, min/d	351.61 (11.34)	349.12 (10.04)	356.72 (16.52)	0.51

Data are shown as weighted percentages (SE).

MASLD, metabolic dysfunction-associated steatotic liver disease; T2DM, type 2 diabetes.

### 3.2 Prevalence of MASLD, MASLD-Related severe steatosis, and significant fibrosis

The prevalence of MASLD in the study cohort was 36.3%. Compared to the non-MASLD population, patients with MASLD were older, more males, and exhibited elevated clinical parameters, including higher BMI, WC, LSM, ALT, TG, TC, HbA1c, and fasting glucose. Additionally, the prevalence of T2DM, hypertension, hyperlipidemia, and obesity were significantly higher in patients with MASLD compared to those without (Table 1).

The prevalence of severe steatosis among patients with MASLD was 72.6%. Compared with patients with non-severe steatosis, those with severe steatosis had significantly higher rates of hypertension, diabetes, and obesity (Supplementary Table S1). Furthermore, 13.4% of the MASLD population had significant fibrosis. The prevalence of hypertension, diabetes, and obesity was also higher in the significant fibrosis group compared to those with early fibrosis (Supplementary Table S2).

### 3.3 Association between sarcopenia and the severity of MASLD

**FIGURE 2:**
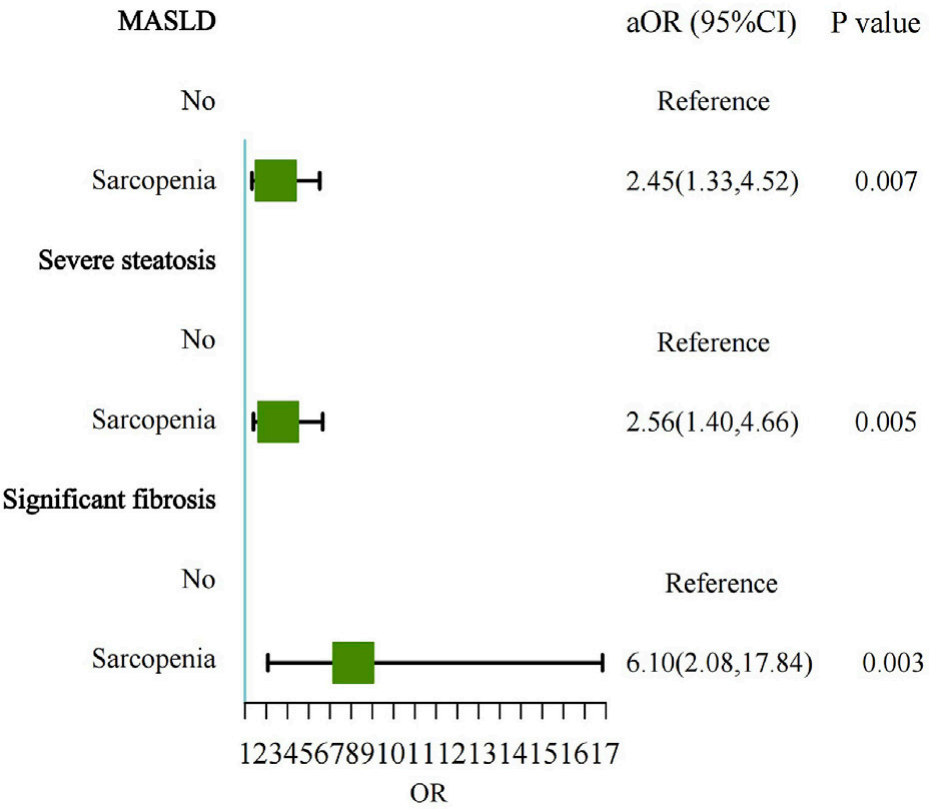
Forest plot showing the multivariable-adjusted risk of sarcopenia for MASLD, severe steatosis, and significant fibrosis. MASLD, Metabolic dysfunction-associated steatotic liver disease; aOR, adjusted odds ratio.

The prevalence of sarcopenia was significantly higher in individuals with MASLD compared to those without MASLD (11.7% vs. 3.8%). The strongest association observed was between sarcopenia and significant fibrosis in MASLD patients. After adjusting for potential confounders, sarcopenia was independently associated with a 6.10-fold increased risk of significant fibrosis (OR = 6.10, 95% CI: 2.08–17.84, P = 0.003) (Table 2; Figure 2). This finding underscores the critical role of sarcopenia in liver fibrosis. Sarcopenia was more prevalent among MASLD patients with significant fibrosis compared to those without significant fibrosis (21.8% vs. 10.0%). Multivariable logistic regression analysis also revealed that sarcopenia was independently associated with a 2.45-fold increased risk of MASLD (OR = 2.45, 95% CI: 1.33–4.52, P = 0.007).

**TABLE 2:** Multivariate ORs of sarcopenia for MASLD, severe steatosis, and significant fibrosis.

Groups	Model 1		Model 2		Model 3	
	OR (95%CI)	P value	OR (95%CI)	P value	OR (95%CI)	P value
Total population, OR for MASLD						
No Sarcopenia	Reference		Reference		Reference	
Sarcopenia	2.81 (1.81, 4.36)	< 0.001	2.55 (1.21, 5.40)	0.033	**2.45 (1.33,4.52)**	**0.007**
Among MASLD, OR for severe steatosis						
No Sarcopenia	Reference		Reference		Reference	
Sarcopenia	2.85 (1.43,5.67)	0.007	2.73 (0.86,8.67)	0.065	**2.56 (1.40,4.66)**	**0.005**
Among MASLD, OR for significant fibrosis						
No Sarcopenia	Reference		Reference		Reference	
Sarcopenia	2.49 (0.89, 6.92)	0.076	2.61 (0.30, 22.99)	0.198	**6.10 (2.08,17.84)**	**0.003**

Model 1was adjusted for age and sex.

Model 2 was adjusted for model 1+ race, education, marital status, smoking.

Model 3 was adjusted for model 2+ T2DM, hypertension, hyperlipidemia, ALT, AST, TG, TC, physical activity.

OR, odds ratio; MASLD, metabolic dysfunction-associated steatotic liver disease.

Bold values indicated p value < 0.05 in the model 3.

Similarly, sarcopenia was more prevalent among individuals with severe steatosis compared to those with mild to moderate steatosis (13.9% vs. 5.4%). In the multivariable model, sarcopenia was independently associated with a 2.56-fold increased risk of severe steatosis (OR = 2.56, 95% CI: 1.40–4.66, P = 0.005).

### 3.4 Association between physical activity and the severity of MASLD

**FIGURE 3:**
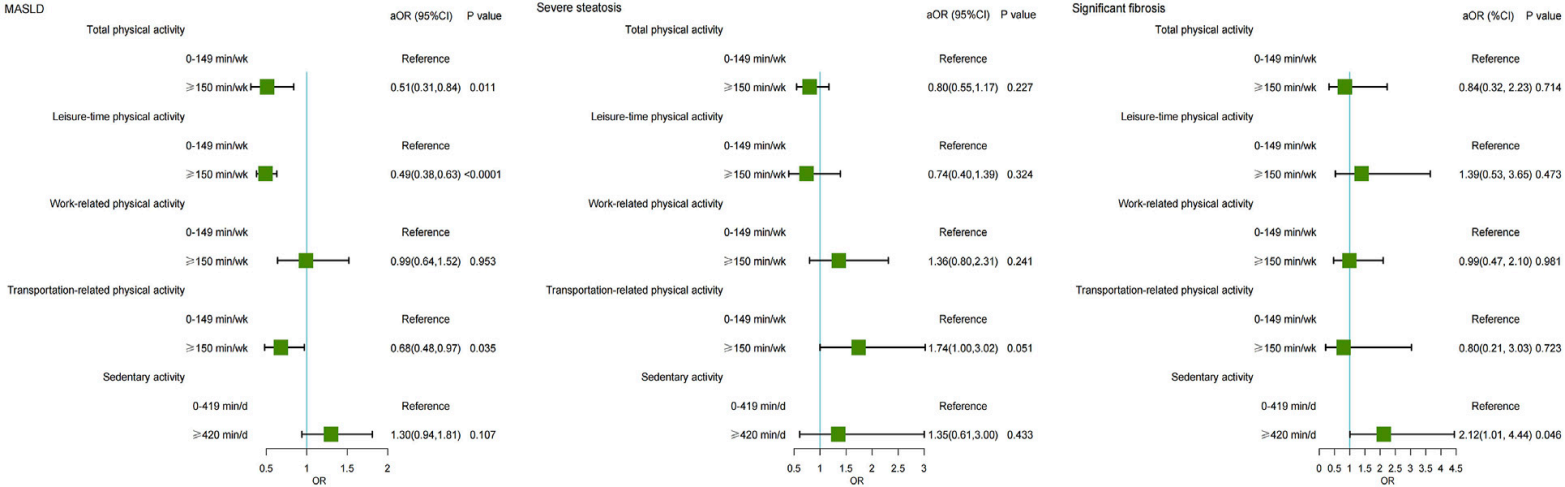
Forest plot showing the multivariable-adjusted risk of physical activity for MASLD, severe steatosis, and significant fibrosis. MASLD, Metabolic dysfunction-associated steatotic liver disease; aOR, adjusted odds ratio.

Higher physical activity was associated with a reduced risk of MASLD. Individuals engaging in total physical activity of ≥150 min/wk had a significantly reduced risk of MASLD (OR = 0.51, 95% CI: 0.31–0.84, P = 0.011). Similarly, individuals participating in ≥150 min/wk of recreational physical activity had a 51% (OR = 0.49, 95% CI: 0.38–0.63, P < 0.001), and those ≥150 min/wk transportation-related physical activity had a 32% (OR = 0.68, 95% CI: 0.48–0.97, P = 0.035) lower risk of MASLD (Table 3). However, no significant association was observed between physical activity and the severity of steatosis. Additionally, those who reported ≥420 min/d of sedentary activity had a 2.12-fold increased risk of significant fibrosis (OR = 2.12, 95% CI: 1.01–4.44, P = 0.046). (Figure 3).

**TABLE 3:** Multivariate ORs of physical activity for MASLD, severe steatosis, and significant fibrosis.

Groups	Model 1		Model 2		Model 3	
	OR (95%CI)	P	OR (95%CI)	P	OR (95%CI)	P
Total population, OR for MASLD						
Total physical activity						
0-149 min/wk	Reference		Reference		Reference	
≥150 min/wk	0.51 (0.32,0.79)	0.006	0.52 (0.20, 1.33)	0.096	**0.51(0.31,0.84)**	**0.011**
Leisure-time physical activity						
0-149 min/wk	Reference		Reference		Reference	
≥150 min/wk	0.52 (0.40,0.67)	<0.001	0.48 (0.28, 0.81)	0.026	**0.49(0.38,0.63)**	**<0.0001**
Work-related physical activity						
0-149 min/wk	Reference		Reference		Reference	
≥150 min/wk	0.95 (0.68,1.32)	0.720	1.05 (0.50, 2.22)	0.804	0.99 (0.64,1.52)	0.953
Transportation-related physical activity						
0-149 min/wk	Reference		Reference		Reference	
≥150 min/wk	0.74 (0.54,1.01)	0.058	0.74 (0.36, 1.51)	0.214	**0.68(0.48,0.97)**	**0.035**
Sedentary activity						
0-419 min/d	Reference		Reference		Reference	
≥420 min/d	1.18 (0.85,1.62)	0.291	1.30 (0.73, 2.32)	0.189	1.30 (0.94,1.81)	0.107
Among MASLD, OR for severe steatosis						
Total physical activity						
0-149 min/wk	Reference		Reference		Reference	
≥150 min/wk	0.86 (0.55,1.33)	0.463	0.89 (0.37,2.16)	0.626	0.80 (0.55,1.17)	0.227
Leisure-time physical activity						
0-149 min/wk	Reference		Reference		Reference	
≥150 min/wk	0.75 (0.45,1.26)	0.246	0.79 (0.25,2.48)	0.473	0.74 (0.40,1.39)	0.324
Work-related physical activity						
0-149 min/wk	Reference		Reference		Reference	
≥150 min/wk	1.53 (0.90,2.61)	0.108	1.46 (0.52,4.13)	0.256	1.36 (0.80,2.31)	0.241
Transportation-related physical activity						
0-149 min/wk	Reference		Reference		Reference	
≥150 min/wk	1.79 (1.18,2.72)	0.011	1.92 (0.84,4.39)	0.076	1.74 (1.00,3.02)	0.051
Sedentary activity						
0-419 min/d	Reference		Reference		Reference	
≥420 min/d	1.40 (0.66,2.98)	0.346	1.50 (0.29,7.65)	0.398	1.35 (0.61,3.00)	0.433
Among MASLD, OR for significant fibrosis						
Total physical activity						
0-149 min/wk	Reference		Reference		Reference	
≥150 min/wk	0.87 (0.33, 2.32)	0.763	0.90 (0.12, 6.51)	0.836	0.84 (0.32, 2.23)	0.714
Leisure-time physical activity						
0-149 min/wk	Reference		Reference		Reference	
≥150 min/wk	1.34 (0.50, 3.60)	0.530	1.48 (0.19,11.48)	0.498	1.39 (0.53, 3.65)	0.473
Work-related physical activity						
0-149 min/wk	Reference		Reference		Reference	
≥150 min/wk	1.13 (0.48, 2.63)	0.763	1.02 (0.24, 4.37)	0.949	0.99 (0.47, 2.10)	0.981
Transportation-related physical activity						
0-149 min/wk	Reference		Reference		Reference	
≥150 min/wk	1.01 (0.35, 2.93)	0.978	1.06 (0.12, 9.63)	0.917	0.80 (0.21, 3.03)	0.723
Sedentary activity						
0-419 min/d	Reference		Reference		Reference	
≥420 min/d	1.83 (0.81, 4.11)	0.129	2.29 (0.46,11.40)	0.156	**2.12(1.01, 4.44)**	**0.046**

Model 1 was adjusted for age and sex.

Model 2 was adjusted for model 1+ race, education, marital status, smoking.

Model 3 was adjusted for model 2 + T2DM, hypertension, hyperlipidemia.

OR, odds ratio; MASLD, metabolic dysfunction-associated steatotic liver disease. Bold values indicated p value <0.05 in the model 3.

### 3.5 Association between sarcopenia, physical activity, and the severity of MASLD

**FIGURE 4:**
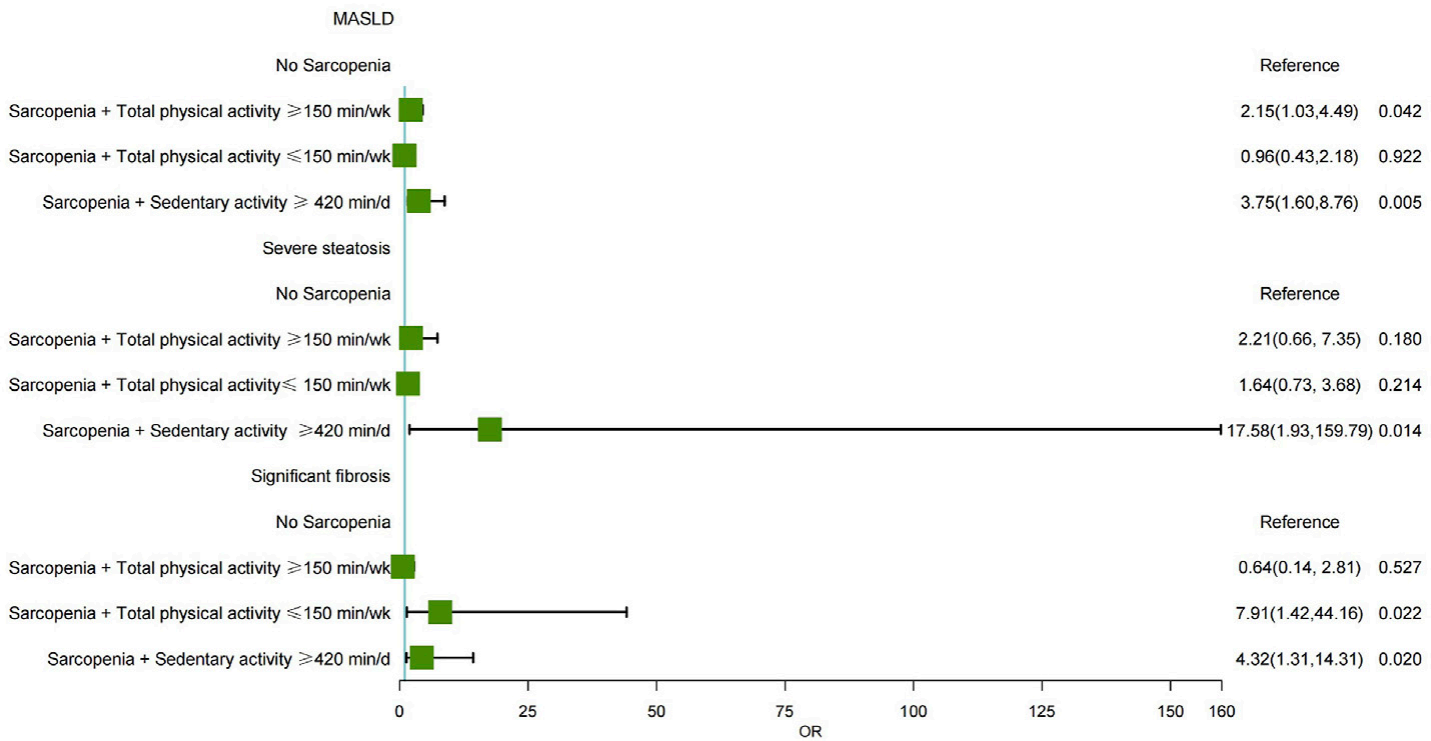
Forest plot showing the multivariable-adjusted risk of sarcopenia and physical activity for MASLD, severe steatosis, and significant fibrosis. MASLD, Metabolic dysfunction-associated steatotic liver disease; aOR, adjusted odds ratio.

Table 4 further investigated the combined effect of sarcopenia and physical activity on the severity of MASLD. Compared to individuals without sarcopenia, those with sarcopenia and high physical activity had a 2.15-fold increased risk of MASLD (OR = 2.15, 95% CI: 1.03–4.49, P = 0.042). Similarly, patients with sarcopenia who reported prolonged sedentary activity (≥420 min/d) exhibited a 3.75-fold increased risk of MASLD (OR = 3.75, 95% CI: 1.60–8.76, P = 0.005). While sarcopenia combined with physical activity showed no statistically significant effect on the severity of steatosis, patients with sarcopenia and prolonged sedentary activity significantly increased the risk of severe steatosis, with an OR of 17.58 (OR = 17.58, 95% CI: 1.93–159.79, P = 0.014). Additionally, compared to those without sarcopenia, individuals with sarcopenia and low physical activity had a 7.91-fold increased risk of developing significant fibrosis (OR = 7.91, 95% CI: 1.42–44.16, P = 0.022). Furthermore, patients with sarcopenia and prolonged sedentary activity had a 4.32 times higher risk of significant fibrosis (OR = 4.32, 95% CI: 1.31–14.31, P = 0.020). (Figure 4).

**TABLE 4:** Multivariate ORs of sarcopenia and physical activity for MASLD, severe steatosis, and significant fibrosis.

Groups	Model 1		Model 2		Model 3	
	OR (95%CI)	P Value	OR (95%CI)	P Value	OR (95%CI)	P Value
OR for MASLD						
No Sarcopenia	Reference		Reference		Reference	
Sarcopenia + Total physical activity ≥150 min/wk	3.44 (1.32, 8.92)	0.017	2.98 (1.30, 6.86)	0.013	**2.15(1.03,4.49)**	**0.042**
Sarcopenia + Total physical activity ≤150 min/wk	1.90 (0.94, 3.84)	0.068	1.75 (0.96, 3.19)	0.066	0.96 (0.43,2.18)	0.922
Sarcopenia + Sedentary activity ≥420 min/d	5.21 (1.88,14.41)	0.005	4.91 (1.94,12.43)	0.002	**3.75(1.60,8.76)**	**0.005**
Among MASLD, OR for severe steatosis						
No Sarcopenia	Reference		Reference		Reference	
Sarcopenia + Total physical activity ≥150 min/wk	2.93 (0.77, 11.23)	0.103	2.72 (0.74, 9.90)	0.120	2.21 (0.66, 7.35)	0.180
Sarcopenia + Total physical activity ≤150 min/wk	1.84 (0.82, 4.11)	0.120	1.64 (0.85, 3.18)	0.133	1.64 (0.73, 3.68)	0.214
Sarcopenia + Sedentary activity ≥420 min/d	19.36 (1.35,277.43)	0.033	22.24 (2.46,201.00)	0.009	**17.58(1.93,159.79)**	**0.014**
Among MASLD, OR for significant fibrosis						
No Sarcopenia	Reference		Reference		Reference	
Sarcopenia + Total physical activity ≥150 min/wk	0.67 (0.27, 1.66)	0.346	0.65 (0.23, 1.80)	0.379	0.64 (0.14, 2.81)	0.527
Sarcopenia + Total physical activity ≤150 min/wk	4.12 (0.95,17.91)	0.057	4.72 (1.04,21.35)	0.045	**7.91(1.42,44.16)**	**0.022**
Sarcopenia + Sedentary activity ≥420 min/d	3.51 (1.33, 9.24)	0.017	3.87 (1.26,11.91)	0.022	**4.32(1.31,14.31)**	**0.020**

Model 1was adjusted for age and sex.

Model 2 was adjusted for model 1+ race, education, marital status, smoking.

Model 3 was adjusted for model 2 + T2DM, hypertension, hyperlipidemia, ALT, AST, TG, TC.

OR, odds ratio; MASLD, metabolic dysfunction-associated steatotic liver disease.Bold values indicated p value <0.05 in the model 3.

### 3.6 Sensitivity analysis

Sensitivity analyses were conducted across various subgroups stratified by sex, age, race, marital status, education level, smoking, hypertension, T2DM, obesity, and hyperlipidemia to assess potential interactions with sarcopenia. Among these factors, only obesity significantly interacted with sarcopenia in the prevalence of MASLD (P = 0.016) (Supplementary Table S3).

## 4 Discussion

Our study highlights a significant association between sarcopenia, physical inactivity, and prolonged sedentary behavior with MASLD progression. While previous studies have demonstrated a significant association between sarcopenia and MASLD, our study is one of the first to explore the combined effects of sarcopenia, physical activity, and sedentary behavior on MASLD progression. Sarcopenic patients who were physical inactive were more likely to have significant fibrosis, while those with prolonged sedentary time were associated with a higher risk of developing and progressing MASLD. This finding suggested the need for targeted interventions to increase physical activity (≥150 min/wk) and reduce sedentary behavior (≤420 min/d) among sarcopenic patients as a potential strategy to mitigate MASLD progression. These findings provide a novel perspective on the importance of addressing both muscle mass and activity levels as part of a comprehensive strategy to manage MASLD.

Previous studies have demonstrated a significant association between sarcopenia and MASLD, which can be attributed to several physiological mechanisms. First, skeletal muscle serves as a primary target tissue for insulin. Reducing muscle mass can exacerbate insulin resistance, leading to increased lipolysis and the accumulation of free fatty acids in the liver, thereby promoting the occurrence and progression of MASLD (Utzschneider and Kahn, 2006). Insulin activates the mammalian target of rapamycin (mTOR), 4E-binding protein 1 (4EBP1), and ribosomal S6 kinase 1 (S6K1). These proteins are involved in protein synthesis, maintenance of muscle mass, and skeletal muscle anabolic metabolism (Fujita et al., 2007). Additionally, elevated levels of chronic inflammation markers, such as interleukin-6 (IL-6) and tumor necrosis factor-alpha (TNF-α), along with reduced secretion of muscle-derived factors, may further contribute to hepatic steatosis and liver inflammation. TNF-α contributed to hepatic lipid accumulation by promoting *de novo* lipogenesis (DNL). It also stimulates nuclear factor kappa B (NF-κB), a key transcription factor for pro-inflammatory cytokines, which facilitates the progression of NAFLD and muscle catabolism (Beyer et al., 2012; Han et al., 2022). Myostatin, a muscle-related protein, has also been implicated in liver fibrosis progression by activating hepatic stellate cells (Kuchay et al., 2022; Miller et al., 2011; Delogu et al., 2019). Myostatin mediates Smad 2/3 activation, which suppresses myogenesis and protein synthesis by inhibiting the Akt-mediated mTOR signaling pathway, ultimately leading to muscle atrophy. Furthermore, it promotes muscle protein degradation through FoxO-dependent activation of the ubiquitin-proteasome pathway and autophagy (Han et al., 2013). In this study, our findings demonstrated that sarcopenia significantly associated with MASLD, severe steatosis, and significant fibrosis, with ORs of 2.45 (95% CI: 1.33–4.52), 2.56 (95% CI: 1.40–4.66), and 6.10 (95% CI: 2.08–17.84), respectively. These results are consistent with previous research, including a meta-analysis of 19 studies that demonstrated a significant association between sarcopenia and NAFLD, NASH, and significant fibrosis, with pooled ORs of 1.33 (95% CI: 1.20-1.48), 2.42 (95% CI: 1.27-3.57), and 1.56 (95% CI: 1.34-1.78), respectively (Cai et al., 2020). These findings further verify the robustness of our results and indicate the potential prevalence of sarcopenia as a critical risk factor in the progression of liver disease. However, the higher OR for significant fibrosis observed in our study. One potential explanation was demographic differences: while the meta-analysis included a mix of older populations and patients with advanced liver disease, our study focused on adults in the United States. Additionally, differences in sarcopenia diagnostic criteria may explain discrepancies; while we used the FNIH definitions, other studies often incorporate muscle strength metrics, such as grip strength, which may influence effect sizes. Our study defined sarcopenia using the FNIH criteria. Specifically, the FNIH criteria primarily emphasize lean mass, which might lead to higher prevalence estimates of sarcopenia in certain populations, particularly younger adults, whereas EWGSOP definitions yield lower prevalence rates due to their focus on functional impairments more characteristic of older populations.

Numerous studies have established a strong association between PA and MASLD. The 2018 American Physical Activity Guidelines recommend that adults engage in at least 150 min/week of moderate-intensity PA, with additional health benefits observed when exceeding 300 min/week (Piercy et al., 2018). PA has beneficial effects on MASLD through various mechanisms, including the activation of uncoupling protein-1, peroxisome proliferator-activated receptor γ (PPAR-γ), adipocytokines, and branched-chain amino acids, which collectively reduce insulin resistance, promote lipolysis, and exert anti-inflammatory and antioxidant effects (Hughes et al., 2021; Babu et al., 2022; Thorp and Stine, 2020; Su et al., 2023). Conversely, sedentary behavior negatively impacts metabolic processes, increasing the risk of health issues such as obesity, diabetes, insulin resistance, and metabolic syndrome (Hamilton et al., 2007). Previous research has shown that engaging in more than 300 min/week of leisure-time PA significantly reduces the association of NAFLD and significant fibrosis in the general population, with ORs of 0.51 (95% CI: 0.40-0.65) and 0.41 (95% CI: 0.22-0.74), respectively. In contrast, sedentary behavior exceeding 8 h/day significantly increases the risk of NAFLD, with an OR of 1.44 (95% CI: 1.01-2.05) (Kim et al., 2022). Additionally, a Korean multicenter study involving 11,690 NAFLD patients found that PA exceeding 600 MET min/week was protective against fibrosis, sarcopenia, and cardiovascular disease. However, fibrosis was assessed using non-invasive serological markers (Chun et al., 2023). Our results indicated that total, leisure-time, and transportation-related PA exceeding 150 min/wk significantly reduce the association with MASLD, while sedentary behavior exceeding 420 min/d increased the association with the development of significant fibrosis in MASLD patients. Notably, our results demonstrated a substantial increase in the risk of severe steatosis among individuals with sarcopenia combined with prolonged sedentary activity, with an odds ratio of 17.58 (95% CI: 1.93–159.79). We acknowledge that the wide confidence interval reflects statistical uncertainty, likely influenced by the small sample size in this subgroup of sarcopenia and sedentary activity ≥420 min/d. However, no significant effect was observed on the risk of severe steatosis. A combined analysis of sarcopenia and physical activity showed that individuals with sarcopenia and prolonged sedentary activity were associated with the development of MASLD and severe fibrosis compared to those without sarcopenia. However, individuals with sarcopenia who engaged in high physical activity were still associated with MASLD, suggesting that while physical activity may decrease the negative effects of sarcopenia, it does not completely balance the elevated risk associated with sarcopenia. Interestingly, individuals with sarcopenia and low-intensity physical activity were associated with a significant association of fibrosis progression in patients with MASLD. These results highlight the need for early identification and management of sarcopenia in patients with MASLD, as well as the promotion of increased physical activity and reduced sedentary behavior. Therefore, it is recommended that individuals with sarcopenia increase their physical activity levels by incorporating both aerobic and resistance exercise. Current guidelines suggest a minimum of 150 min of moderate-intensity aerobic exercise per week, along with resistance training based on established exercise prescription principles, which should target major muscle groups at least 1-2 times per week. Additionally, progressive resistance exercises, tailored to the individual’s capacity, can help improve muscle mass and strength, while aerobic exercises enhance cardiovascular health and metabolic function.

This study had several limitations that should be considered when interpreting the findings. First, the NHANES data used in this study were cross-sectional, limiting our ability to infer causality. Second, physical activity and sedentary time were assessed via self-reported data using the GPAQ, which may introduce recall and reporting biases. Third, the study population was restricted to individuals aged 18-59 years, while sarcopenia is more prevalent in older adults aged 60 years and above. Fourth, there were more recent definitions of sarcopenia (e.g., EWGSOP, AWGS), the FNIH criteria were specifically used in this study because there is no indicator of grip strength in the NHANES data. Fifth, while the study sample is representative of the United States population, the small sample size of certain ethnic groups may affect the generalizability of the results to these populations. Sixth, DXA-derived ALM adjusted for BMI, as applied using the FNIH criteria, may overestimate muscle mass in obese individuals. Finally, despite adjusting for multiple covariates, the possibility of residual confounding by unmeasured variables cannot be entirely ruled out.

## 5 Conclusion

In conclusion, the study emphasized the pivotal role of sarcopenia and physical activity in the progression of MASLD. Sarcopenia is associated with an increased risk of severe steatosis and significant fibrosis, while physical activity demonstrates a protective effect against MASLD, with a more limited impact on disease severity. These findings underscore the importance of early screening for sarcopenia in patients with MASLD, as timely targeting interventions to improve muscle mass and metabolic health. From a prevention perspective, integrating exercise programs that combine resistance and aerobic exercise into routine clinical practice could benefit patients with MASLD, particularly those with sarcopenia. Such interventions not only improve muscle strength and function but also mitigate the synergistic effects of sarcopenia and sedentary behavior in promoting liver fibrosis. Further longitudinal studies are warranted to explore the mechanisms underlying these associations and to identify effective strategies for mitigating the impact of sarcopenia and inactivity in MASLD management.

## Data Availability

The datasets presented in this study can be found in online repositories. This data can be found here: https://wwwn.cdc.gov/nchs/nhanes/Default.aspx.
