# Supplementary material for: Association of sarcopenia and physical activity on the severity of metabolic dysfunction-associated steatotic liver disease among United States adults: NHANES 2017 - 2018
Source: Front Aging. 2025 May 13;6:1573170. doi: 10.3389/fragi.2025.1573170 (PMC12106442; doi:10.3389/fragi.2025.1573170)
Supplement: Supplementary file 1 [file Table1.docx]

**Supplementary Tables**

Supplementary Table 1. Baseline characteristics of participants according to the severe steatosis in patients with MASLD.

| Variables | Total (n=664) | Non- Severe steatosis (n=182) | Severe steatosis (n=482) |  |
| --- | --- | --- | --- | --- |
| Age, y |  |  |  | 0.41 |
| 18-29 | 17.80(0.02) | 14.06(2.88) | 19.14(2.42) |  |
| 30-44 | 30.99(0.03) | 31.48(4.16) | 30.82(2.63) |  |
| 45-60 | 51.21(0.06) | 54.46(4.01) | 50.04(3.13) |  |
| Male, % | 58.51(0.06) | 51.92(6.86) | 60.88(2.29) | 0.26 |
| Race, % |  |  |  | 0.07 |
| Mexican American | 13.03(0.03) | 10.14(3.23) | 14.07(3.36) |  |
| Asian | 11.12(0.02) | 13.79(3.84) | 10.15(2.08) |  |
| Non-Hispanic Black | 9.80(0.01) | 10.52(1.88) | 9.54(1.83) |  |
| Non-Hispanic White | 53.28(0.07) | 48.19(5.41) | 55.12(4.45) |  |
| Other Hispanic | 7.93(0.01) | 7.52(2.31) | 8.08(1.65) |  |
| Other Race | 4.84(0.02) | 9.85(4.51) | 3.04(0.78) |  |
| College education, % | 63.12(0.06) | 69.36(5.35) | 60.87(3.31) | 0.11 |
| Married, % | 63.81(0.06) | 63.69(2.73) | 63.86(3.57) | 0.97 |
| Smoke, % |  |  |  | 0.41 |
| Former | 23.23(0.04) | 19.50(4.94) | 24.58(3.54) |  |
| Never | 66.36(0.05) | 67.08(7.32) | 66.11(4.02) |  |
| Now | 10.40(0.02) | 13.43(3.41) | 9.32(1.73) |  |
| Body mass index, kg/m^2^ | 32.46(0.46) | 29.00(0.68) | 33.71(0.47) | < 0.0001 |
| Waist circumference, cm | 107.25(1.18) | 99.56(1.52) | 110.02(1.20) | < 0.0001 |
| Liver stiffness measurement, kPa | 6.10(0.23) | 4.76(0.16) | 6.59(0.29) | < 0.0001 |
| Alanine aminotransferase, IU/L | 26.94(0.98) | 21.98(1.39) | 28.62(0.90) | < 0.0001 |
| Aspartate aminotransferase, IU/L | 22.09(0.58) | 20.04(0.98) | 22.78(0.59) | 0.01 |
| Triglyceride, mmol/L | 2.11(0.11) | 1.93(0.22) | 2.17(0.12) | 0.31 |
| Total cholesterol, mmol/L | 4.97(0.06) | 5.09(0.11) | 4.93(0.07) | 0.19 |
| Fasting glucose, mg/dL | 106.08(2.96) | 98.23(2.56) | 108.75(3.60) | 0.02 |
| Hemoglobin A1c, % | 5.86(0.08) | 5.64(0.11) | 5.93(0.09) | 0.03 |
| Sarcopenia, % | 11.65(0.02) | 5.35(1.34) | 13.92(2.47) | 0.004 |
| Hypertension, % | 39.41(0.05) | 25.99(5.23) | 44.24(3.21) | 0.02 |
| T2DM, % | 28.15(0.04) | 17.35(4.36) | 32.05(3.32) | 0.02 |
| Hyperlipidemia, % | 74.24(0.06) | 71.73(6.39) | 75.14(2.22) | 0.60 |
| Obesity, % | 67.03(0.08) | 44.47(6.76) | 75.15(3.71) | < 0.0001 |
| Total physical activity, min/wk | 1339.52(113.66) | 1271.31(236.85) | 1364.38(112.01) | 0.70 |
| Work-related physical activity, min/wk | 768.44(106.36) | 757.20(187.10) | 772.49 (99.18) | 0.93 |
| Transportation-related physical activity, min/wk | 63.65(10.21) | 32.87 (5.85) | 74.74(13.99) | 0.02 |
| Leisure-time physical activity, min/wk | 213.81(19.70) | 211.09(23.98) | 214.79(24.16) | 0.91 |
| Sedentary activity, min/d | 356.72(16.52) | 316.33(28.08) | 371.15(15.82) | 0.05 |

Data are shown as weighted percentages (SE).

MASLD, metabolic dysfunction-associated steatotic liver disease; T2DM, type 2 diabetes.

Supplementary Table 2. Baseline characteristics of participants according to the significant fibrosis in patients with MASLD.

| Variables | Total (n=664) | Non-fibrosis (n=575) | Fibrosis (n=89) | P value |
| --- | --- | --- | --- | --- |
| Age, y | 42.80(0.69) | 42.02(0.81) | 47.66(1.39) | 0.02 |
| 18-29 | 17.80(0.02) | 19.83(1.98) | 5.25(1.95) |  |
| 30-44 | 30.99(0.03) | 32.34(2.98) | 22.67(5.81) |  |
| 45-60 | 51.21(0.06) | 47.83(3.83) | 72.08(6.29) |  |
| Male, % | 58.51(0.06) | 59.06(2.75) | 55.13(7.39) | 0.66 |
| Race, % |  |  |  | 0.24 |
| Mexican American | 13.03(0.03) | 13.63(3.22) | 9.33(3.35) |  |
| Asian | 11.12(0.02) | 11.68(2.47) | 7.65(1.90) |  |
| Non-Hispanic Black | 9.80(0.01) | 9.43(1.26) | 12.10(3.80) |  |
| Non-Hispanic White | 53.28(0.07) | 52.40(4.26) | 58.71(6.49) |  |
| Other Hispanic | 7.93(0.01) | 7.65(1.46) | 9.61(2.00) |  |
| Other Race | 4.84(0.02) | 5.21(1.76) | 2.60(1.06) |  |
| College education, % | 63.12(0.06) | 64.10(3.40) | 57.04(10.67) | 0.53 |
| Married, % | 63.81(0.06) | 63.33(2.66) | 66.81(5.90) | 0.49 |
| Smoke, % |  |  |  | 0.64 |
| Former | 23.23(0.04) | 23.50(3.40) | 21.58(7.63) |  |
| Never | 66.36(0.05) | 65.59(4.09) | 71.14(7.60) |  |
| Now | 10.40(0.02) | 10.91(2.12) | 7.28(2.25) |  |
| Body mass index, kg/m^2^ | 32.46(0.46) | 31.69(0.48) | 37.24(1.05) | < 0.001 |
| Waist circumference, cm | 107.25(1.18) | 105.23(1.18) | 119.82(2.46) | < 0.0001 |
| Liver stiffness measurement, kPa | 6.10(0.23) | 4.90(0.07) | 13.55(1.18) | < 0.0001 |
| Alanine aminotransferase, IU/L | 26.94(0.98) | 25.99(1.03) | 32.65(2.47) | 0.02 |
| Aspartate aminotransferase, IU/L | 22.09(0.58) | 21.49(0.59) | 25.65(1.49) | 0.02 |
| Triglyceride, mmol/L | 2.11(0.11) | 2.02(0.10) | 2.67(0.48) | 0.19 |
| Total cholesterol, mmol/L | 4.97(0.06) | 4.97(0.06) | 4.92(0.19) | 0.79 |
| Fasting glucose, mg/dL | 106.08(2.96) | 102.39(2.18) | 128.23(7.81) | 0.002 |
| Hemoglobin A1c, % | 5.86(0.08) | 5.73(0.07) | 6.66(0.19) | < 0.001 |
| Sarcopenia, % | 11.65(0.02) | 10.01(1.78) | 21.76(7.37) | 0.08 |
| Hypertension, % | 39.41(0.05) | 35.63(2.64) | 62.76(8.12) | 0.004 |
| T2DM, % | 18.60(0.03) | 14.79(2.48) | 42.07(7.04) | < 0.0001 |
| Hyperlipidemia, % | 74.24(0.06) | 73.21(3.11) | 80.55(6.12) | 0.4 |
| Obesity, % | 67.03(0.08) | 63.36(4.64) | 89.66(4.95) | 0.01 |
| Total physical activity, min/wk | 1339.52(113.66) | 1391.83(117.05) | 996.03(168.37) | 0.02 |
| Work-related physical activity, min/wk | 768.44(106.36) | 812.67(109.82) | 495.54(150.39) | 0.04 |
| Transportation-related physical activity, min/wk | 63.65(10.21) | 66.09(11.55) | 48.56(13.08) | 0.32 |
| Leisure-time physical activity, min/wk | 213.81(19.70) | 217.22(21.15) | 192.81(58.47) | 0.71 |
| Sedentary activity, min/d | 356.72(16.52) | 351.75(15.70) | 387.02(31.76) | 0.20 |

Data are shown as weighted percentages (SE).

MASLD, metabolic dysfunction-associated steatotic liver disease; T2DM, type 2 diabetes.

Supplementary Table 3. Sensitivity Analysis for MASLD.

| **Variables** | OR (95%CI) | P value | P for interaction |
| --- | --- | --- | --- |
| **Age** |  |  | 0.158 |
| 18-29 | 3.82(1.86,7.88) | 0.001 |  |
| 30-44 | 4.83(1.64,14.22) | 0.007 |  |
| 45-60 | 1.90(1.08,3.33) | 0.029 |  |
| **Sex** |  |  | 0.756 |
| Male | 3.63(1.44,9.15) | 0.010 |  |
| Female | 2.90(1.25,6.72) | 0.017 |  |
| **Hypertension** |  |  | 0.127 |
| No | 3.55(2.22,5.66) | <0.0001 |  |
| Yes | 1.79(0.81,3.97) | 0.140 |  |
| **T2DM** |  |  | 0.667 |
| No | 2.61(1.53,4.46) | 0.002 |  |
| Yes | 3.48(1.10,10.96) | 0.035 |  |
| **Hyperlipidemia** |  |  | 0.352 |
| No | 3.62(1.67,7.82) | 0.003 |  |
| Yes | 2.39(1.46,3.91) | 0.002 |  |
| **Race** |  |  | 0.296 |
| Non-Hispanic Asian | 2.78(1.20,6.49) | 0.021 |  |
| Mexican American | 2.91(1.25,6.75) | 0.019 |  |
| Non-Hispanic Black | 24.78(3.69,166.42) | 0.003 |  |
| Non-Hispanic White | 2.65(1.25,5.64) | 0.015 |  |
| Other Hispanic | 4.46(1.50,13.27) | 0.012 |  |
| Other Race | 1.69(0.37,7.80) | 0.475 |  |
| **Obesity** |  |  | **0.013** |
| No | 3.88(1.87,8.05) | 0.001 |  |
| Yes | 1.09(0.59,2.02) | 0.769 |  |
| **Military** |  |  | 0.953 |
| No | 3.64(1.97,6.72) | <0.001 |  |
| Yes | 3.72(2.14,6.49) | <0.001 |  |
| **Education** |  |  | 0.766 |
| No | 3.39(2.10,5.48) | <0.0001 |  |
| Yes | 3.84(1.83,8.04) | 0.002 |  |
| **Smoke** |  |  | 0.523 |
| Never | 3.48(1.93,6.30) | <0.001 |  |
| Former | 2.10(0.45,9.72) | 0.318 |  |
| Now | 5.82(1.66,20.48) | 0.009 |  |

MASLD, metabolic dysfunction-associated steatotic liver disease; T2DM, type 2 diabetes.
